# Routine clozapine assay monitoring to improve the management of treatment-resistant schizophrenia

**DOI:** 10.1192/bjb.2021.36

**Published:** 2022-10

**Authors:** David Kitchen, Alex Till, Panchu Xavier

**Affiliations:** 1 Mersey Care NHS Foundation Trust, Liverpool, UK; 2 Mersey Internal Audit Agency, Liverpool, UK; 3 Health Education England (North West), Liverpool, UK

**Keywords:** Clozapine, treatment-resistant schizophrenia, assay monitoring, therapeutic drug monitoring, mortality

## Abstract

**Aims and method:**

Routine therapeutic drug monitoring in clozapine therapy has previously not been considered justifiable. Using observational data, the clinical utility of annual clozapine assay monitoring is explored within a large mental health trust.

**Results:**

After the introduction of routine monitoring, the rate of clozapine assays rose to 2.3 per patient per year, with a consistent reduction in high-risk clozapine assays (<0.1 mg/L or >1.0 mg/L or any result more than 24 months old). High-risk assays are associated with a mortality rate of 31.6 deaths per 1000 patients, more than twice that of those within the target range (0.35–0.60 mg/L and conducted within the past 12 months) (*P* = 0.048).

**Clinical implications:**

Routine clozapine assay monitoring has significant clinical utility. Our simple but targeted approach can be readily implemented to reduce the number of patients with high-risk clozapine assay levels, potentially reduce all-cause mortality and provide optimal treatment for those with treatment-resistant schizophrenia.

Clozapine is the gold standard and only licensed treatment available for treatment-resistant schizophrenia. It is bioequivalent across the available licensed brands^1^ and comparative clozapine serum and plasma assay concentrations are insignificant clinically.^2^

Therapeutic drug monitoring is not mandatory in any country^3^ and, following the initial titration period, the longer-term clinical utility of routine clozapine assay monitoring has been questioned,^4–6^ with limited manufacturers’ guidance available.^7^ In the UK, only 50–60% of patients prescribed clozapine for more than 1 year have clozapine assay levels performed within 12 months.^8^

This article explores the introduction of routine therapeutic drug monitoring as a clinical tool to reduce high-risk therapeutic drug levels, support individualised therapeutic regimes and improve the management of clozapine therapy in treatment-resistant schizophrenia.

## Method

All patients prescribed clozapine were identified across acute, community, low and medium secure mental health services within a large mental health provider in the UK that covers a core weighted population of 1.1 million individuals.

Clozapine assay sampling was uncontrolled and conducted over the course of routine clinical practice. Results were directly obtained from Viapath pathology services and independently analysed by the authors.

Four predetermined clozapine assay risk categories were established pragmatically, based on clinical utility and established consensus:^6^

- Group 1: Never conducted
- Group 2: Target range (0.35–0.60 mg/L and conducted in the past 12 months)
- Group 3: Low risk (0.1–0.35 mg/L or 0.6–1.00 mg/L and conducted in the past 24 months)
- Group 4: High risk (<0.1 mg/L or >1.00 mg/L or any result conducted more than 24 months ago).

The frequency of clozapine assay monitoring and total number of patients prescribed clozapine were monitored from 1 January 2007 to 30 June 2020, with point prevalence surveys taken in 2011 and annually from 2015 onwards.

Patients with a diagnosis of schizophrenia (*n* = 780) or schizoaffective disorder (*n* = 94) were subclassified, with their clozapine adherence reviewed over a 5-year period from 1 July 2015 to 30 June 2020. Patients’ rationale for discontinuing clozapine was identified from their electronic patient record, and ongoing adherence confirmed through active blood monitoring service registration and enrolment with the trust's clozapine dispensary.

All patients with a diagnosis of schizophrenia or schizoaffective disorder between 1 January 2011 and 30 June 2020 who were prescribed clozapine at the time of death or who died within 6 months of clozapine discontinuation were included for mortality analysis. Patients’ most recent clozapine assay could only be reliably cross-referenced between 2015 and 2019.

Ethics board approval and informed consent were not required for this study.

## Results

### Frequency of clozapine assay monitoring

**Fig. 1:**
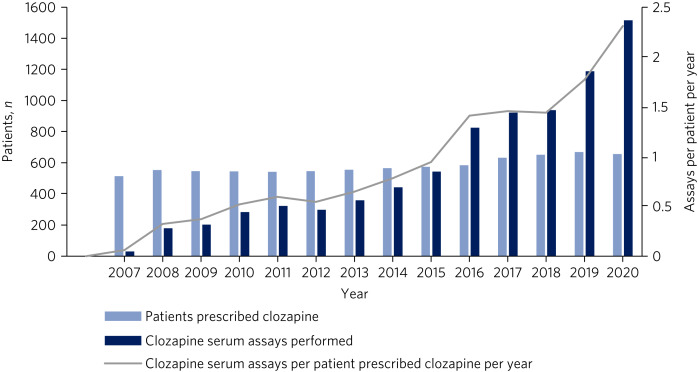
Annual frequency of clozapine assays cross-referenced against the total number of clozapine assays performed and patients prescribed clozapine from 1 January 2007 to 30 June 2020.

The frequency of clozapine assay monitoring has risen beyond the increase in clozapine prescriptions. As identified in Fig. 1, the clozapine assay monitoring rate has risen from 0.1 assays per patient per year in 2007 to 2.3 assays per patient per year projected at the end of 2020, following the implementation of a ‘clozapine prescribing and monitoring policy’ in the trust in 2016.

### Clozapine assay risk categories

Prior to the introduction of the clozapine prescribing and monitoring policy, 66% of patients had never had a clozapine assay performed and only 12% of monitored patients had a clozapine assay within the target therapeutic range.

**Fig. 2:**
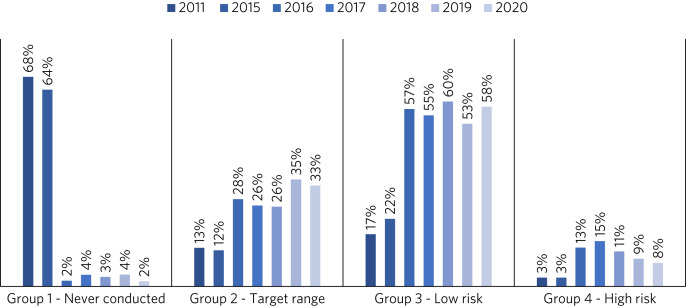
Distribution of patients in the clozapine assay risk categories before and after the implementation of a clozapine prescribing and monitoring policy in the trust in 2016.

However, as highlighted in Fig. 2, clozapine assay monitoring has subsequently increased. Following an initial spike in the high-risk group as they were identified for the first time, this group has steadily declined, accounting for only 8% of patients in 2020.

There has similarly been a corresponding increase in the number of patients whose levels fall within the target or low-risk range (averaging 86% combined between 2016 and 2020), leaving the 2% of patients (*n* = 13) in 2020 who have never been tested, being almost exclusively those undergoing their initial titration.

### Duration of clozapine therapy

In total, 874 patients with schizophrenia or schizoaffective disorder were prescribed clozapine between 1 July 2015 and 30 June 2020, equating to 79.5 individuals being prescribed clozapine per 100 000 people within our population.

Of these, 72.1% (*n* = 630) were prescribed clozapine throughout this period (with an average 9.6-year duration of clozapine adherence); 7.7% (*n* = 67) were transferred to another provider and therefore their duration of adherence is unknown; 14.5% (*n* = 127) had clozapine discontinued (with an average 4-year duration of adherence); and 5.7% (*n* = 50) died (with an average 10.7-year duration of adherence).

Of the 14.5% (*n* = 127) for whom clozapine was discontinued, 6% (*n* = 8) did not achieve a satisfactory therapeutic response to clozapine; 34% (*n* = 43) did not tolerate clozapine; 44% (*n* = 56) did not adhere to the clozapine regime; 13% (*n* = 17) received a red alert for clozapine-induced neutropenia or agranulocytosis; and 2% (*n* = 3) took an overdose on clozapine.

### All-cause mortality cross-referenced with clozapine assay monitoring

In total, 91 patients (average age of 53 years) prescribed clozapine for a diagnosis of schizophrenia or schizoaffective disorder died between 1 January 2011 and 30 June 2020, giving a mortality rate of 15 per 1000 patients; 35% (*n* = 32) died with therapeutic clozapine assay levels and 65% (*n* = 59) with levels outside of the identified therapeutic range or never having had a clozapine assay conducted.

**Fig. 3:**
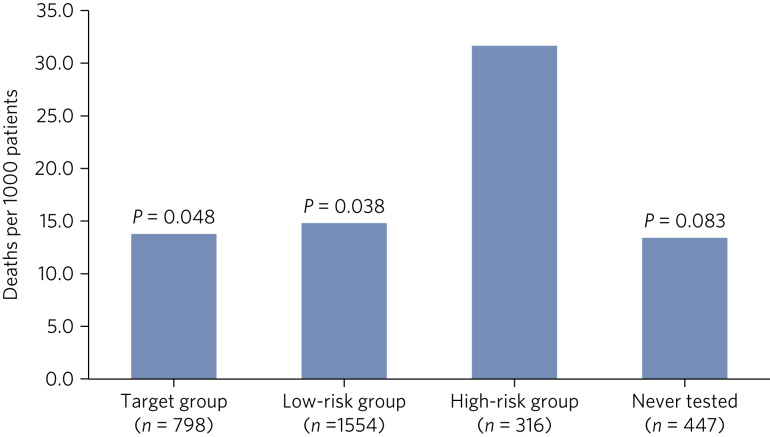
Mortality rate per 1000 patients throughout 2015–2019 across the four clozapine assay risk categories, with *P*-values in relation to the high-risk group.

Mortality rates per 1000 patients were available between 2015 and 2019, with Fig. 3 highlighting significant differences. The high-risk group accounted for the highest mortality rate, at 31.6 deaths per 1000 patients, more than twice that of any other group, with 58% having a clozapine assay level >1.0 mg/L.

Comparing overall mortality before and after the introduction of the clozapine prescribing and monitoring policy (2011–2015 versus 2016–2019), we observed a very modest overall reduction in mortality, from 16.5 to 15.4 deaths per 1000, and a generally reducing trend over the 8-year duration studied.

## Discussion

Higher clozapine assay levels expose individuals to greater risk of dose-related adverse effects,^9^ and although clozapine therapy in itself is thought to reduce all-cause mortality,^10,11^ there is a lack of evidence specifically considering the relationship between clozapine assay levels and mortality.

Through the implementation of a clozapine prescribing and monitoring policy, where clozapine assay monitoring was recommended at least annually, the trust grew to perform one of the highest frequencies of clozapine assay levels per annum for patients who have been prescribed clozapine for over 12 months.^7^

With routine clozapine assay monitoring, there has been a consistent reduction in the number of patients with high-risk clozapine assay levels and an increase in the number with levels within the target range. This is reassuring, because the mortality rate in the high-risk group is over twice the mortality rate for other groups, and an overall very modest reduction in all-cause mortality has been observed.

### Limitations

This study was limited by the uncontrolled observational nature of its design, with data collection based on clinical interest over the course of routine clinical practice. Confounding factors influencing intra- and inter-individual clozapine assay variability were therefore unmeasured (with sample timing and smoking status likely to have the greatest effect). However, these findings remain novel and of significant clinical interest. In particular, there was an apparent rise in local prescribing confidence, as clozapine therapy increased 3% above the national average (9 *v.* 6%) between 2012 and 2018.^12,13^

Furthermore, of the 14.5% of patients for whom clozapine was discontinued, in 44% of these cases this was attributable to non-adherence. This supports findings elsewhere where it has been suggested that the fear of poor adherence may too commonly be used as a reason for not commencing clozapine^13^ and that clozapine has been significantly associated with lower rates of all-cause discontinuation compared with other oral second-generation antipsychotics.^14^

### Clinical implications

Our analysis is one of the most comprehensive datasets of clozapine assay levels published to date. We highlight that routine clozapine assay monitoring in clozapine therapy has significant clinical utility for the well-educated clinician. It can reduce the proportion of patients with high-risk clozapine assay levels, improve the under-utilisation of clozapine therapy by improving prescriber confidence and, of significant clinical interest, can potentially contribute to reduced all-cause clozapine mortality.

## Data Availability

The data that supports the findings of this study are available on reasonable request from the corresponding author (D.K.). The data are not publicly available due to their containing information that could compromise patient confidentiality.

## About the authors

**David Kitchen**, BPharm, MSc, is Senior Clinical Pharmacist (Education and Training) with Mersey Care NHS Foundation Trust, and an associate consultant with Mersey Internal Audit Agency (NHS), Liverpool, UK. **Alex Till**, MBChB, MRCPsych, MSc, MBA, is a specialty registrar in forensic psychiatry with Health Education England (North West), Manchester, UK. **Panchu F. Xavier**, MBBS, PG Cert, MRCPsych, MSc, is Deputy Medical Director for Quality and Patient Safety with Mersey Care NHS Foundation Trust, Liverpool, UK.
